# Characterizing Pressure-Induced Uranium C=H Agostic Bonds**

**DOI:** 10.1002/anie.201411250

**Published:** 2015-04-16

**Authors:** Polly L Arnold, Alessandro Prescimone, Joy H Farnaby, Stephen M Mansell, Simon Parsons, Nikolas Kaltsoyannis

**Affiliations:** EaStCHEM School of Chemistry, University of EdinburghThe King's Buildings, Edinburgh, EH9 3FJ (UK); Department of Chemistry, University College London20 Gordon Street, London, WC1H 0AJ (UK); Current address: Department of Chemistry, University of BaselSpitalstrasse 51, 4056 Basel (Switzerland); Current address: Department of Chemistry, Imperial College LondonSouth Kensington Campus, London, SW7 2AZ (UK); Current address: Institute of Chemical Sciences, Heriot-Watt UniversityEdinburgh, EH14 4AS (UK)

**Keywords:** actinides, density functional calculations, high-pressure chemistry, uranium, X-ray diffraction

## Abstract

The diuranium(III) compound [UN′′_2_]_2_(μ-η^6^:η^6^-C_6_H_6_) (N′′=N(SiMe_3_)_2_) has been studied using variable, high-pressure single-crystal X-ray crystallography, and density functional theory. In this compound, the low-coordinate metal cations are coupled through π- and δ-symmetric arene overlap and show close metal=CH contacts with the flexible methyl CH groups of the sterically encumbered amido ligands. The metal–metal separation decreases with increasing pressure, but the most significant structural changes are to the close contacts between ligand CH bonds and the U centers. Although the interatomic distances are suggestive of agostic-type interactions between the U and ligand peripheral CH groups, QTAIM (quantum theory of atoms-in-molecules) computational analysis suggests that there is no such interaction at ambient pressure. However, QTAIM and NBO analyses indicate that the interaction becomes agostic at 3.2 GPa.

Simple, low-coordinate U^III^ complexes have been recently reported to show a rich reactivity with inert small molecules.[1] This reactivity is despite the fact that in the solid state, structural studies show close contacts between the metal and peripheral ligand C and H atoms that sometimes protect a potential coordination site and sometimes simply block further reactions. For example, hydrocarbon solutions of [U(N′′)_3_] (N′′=N(SiMe_3_)_2_)[2] (containing three U=C_SiMe3_ close contacts, average distance=3.047 Å) reductively couple CO,[3] whereas those of [U{N(SiMe_2_Ph)_2_}_3_] (containing three U=C_ipsoPh_ close contacts, average distance=3.093 Å)[4] do not.

Arguably the most useful homogeneous C=H bond functionalization reactions currently being developed for d-block metal catalysts rely on CH metallation of a bound substrate.[5] Similarly, U complexes that show CH metallation[6] have been developed into catalysts for N-heterocycle coupling, for example.[7] The U^III^ aryloxide [{(ArO)_3_tacn}U(*c*C_6_H_12_)] (tacn=1,4,7-triazacyclonane) shows an intermolecular C=H contact to a molecule of cyclohexane solvent with a U=C distance of 3.864(7) Å and an η^2^-CH interaction suggested by calculations.[8] More generally, agostic interactions between uranium centers and ligand CH groups are often invoked from inspection of close metal–ligand contacts in X-ray structures.[9] No routine method exists for assessing the strength and influence of weak interactions to confirm a genuine agostic interaction in paramagnets, where traditional NMR spectral methods do not work[10] and neutron diffraction studies are not readily available.[11] Computationally, the electron-density-based quantum theory of atoms-in-molecules (QTAIM), with its simple definition of a chemical bond, has been successfully employed to identify agostic interactions.[12]

Systems in which energetically competitive structures are related by subtle changes, such as agostic interactions, may be sensitive to external conditions such as pressure. High pressure has previously shown unconventional behavior in coordination compounds, such as switching the direction of the Jahn–Teller axis in [CuF_2_(H_2_O)_2_(pyrazine)]_*n*_,[13] slowing magnetic relaxation in the single molecule magnet [Mn_12_O_12_(O_2_CCH_2_*t*Bu)_16_(H_2_O)_4_].CH_2_Cl_2_.MeNO_2_,[14] and increasing the coordination number of Cu^II^ in [HGu][Cu_2_(OH)(citrate)(Gu)_2_] (Gu=guanidine)[15] and Pd^II^ in *cis*-[PdCl_2_([9]aneS_3_)] ([9]aneS_3_=1,4,7-trithiacyclononane).[16] High pressure structures of actinide materials have yielded fundamental information on strongly correlated f electrons,[17] but the effect on organoactinide complexes with soft organic ligands has not been investigated.

The U_2_(μ-arene) motif has been observed in a variety of complexes and assigned a range of metal formal oxidation states and levels of ring reduction, suggesting a shallow potential energy surface.[18] We considered that the softness of this uranium–arene bonding interaction and the existence of close U⋅⋅⋅CH interactions in the solid state in [UN′′_2_]_2_(μ-C_6_H_6_) (**1**; Figure 1), recently reported by us,[18g] makes it an interesting target to study in the solid state. Herein, we report the effect of pressure on the molecular and electronic structure. We were keen to explore the possibility that pressure induces agostic binding regimes, and have assessed this computationally using the QTAIM and natural bond orbital (NBO) approaches.

**Figure 1:**
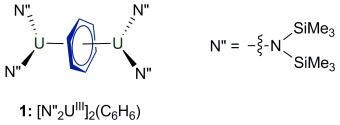
Complex 1.

The structure of **1** was determined at pressures[19] up to 3.2 GPa. The first, perhaps obvious, effect of pressure is the decrease of the unit-cell volume by 15 % at 3.20 GPa (Tables S2–4 in the Supporting Information). At 1.8 GPa **1** undergoes a phase transition which decreases the crystallographic symmetry from *P*2_1_*/c* to *P*$\bar 1$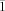, producing two independent molecules in the asymmetric unit: one is not modified further but in the other there is a slight shortening of the U⋅⋅⋅U distance from 4.2492(2) Å to 4.191(5) Å at 3.2 GPa.

Of greater interest is the effect of pressure on U=CH contacts. C4 and C10, atoms on methyl groups in the amido ligands, move closer to the U center, for example with the U1=C10 distance shortening from 3.022(3) Å at ambient pressure to 2.95(2) Å at 3.2 GPa (Figure 2, Table 1). A close intermolecular contact between the C10 and C6 atoms of neighbouring molecules of **1** of 4.03(5) Å at 0 GPa also decreases to 3.48(7) Å at 3.2 GPa. Although this is not the shortest intermolecular contact (the distance from a Me group to a bridging arene shortens from 3.666(5) Å to 3.24(5) Å), it demonstrates a flexibility in the sterically unsaturated molecules that could correlate with the decreasing intramolecular C10=U1 distance. Such short contacts demonstrate the effect of shrinking the cell volume in a structure without obvious cavities.

**Figure 2:**
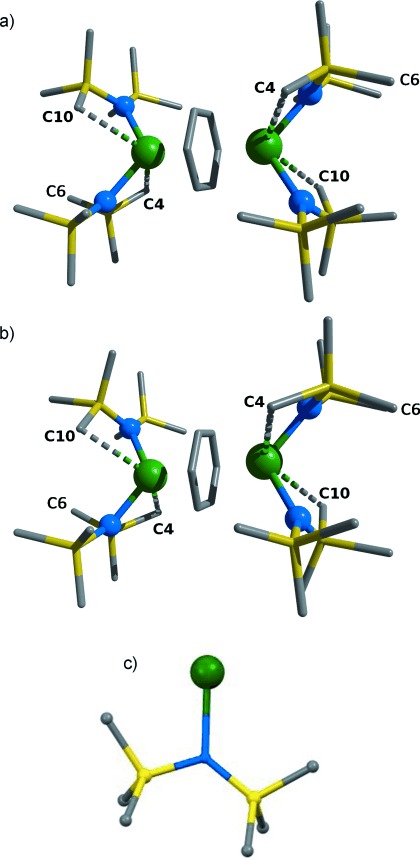
Structure of 1 at a) ambient pressure and b) 3.20 GPa. Asymmetry in the N′′ ligand is shown in (c). H atoms omitted for clarity. Atom colors: green=U; gold=Si; blue=N; gray=C.

**Table 1:** Selected distances as function of pressure for 1.[a]

P [GPa]	U1=U1′ [Å]	U1=C4 [Å]	U1=C10 [Å]	
0.0	4.2492(2)	3.025(3)	3.022(3)	
0.8	4.2367(8)	3.023(13)	3.038(12)	
1.3	4.226(2)	2.98(3)	3.00(3)	
1.8	4.206(4)	3.01(4)	3.00(2)	
2.3	4.202(5)	2.94(4)	2.98(2)	
3.2	4.191(5)	3.00(5)	2.95(2)	

[a] Distances are reported for one of the two independent molecules in the asymmetric unit.

The average uranium–carbon single bond in the Cambridge Structural Database is 2.48 Å, but a variety of longer, formally single or double U=C bonds, have also been reported. Examples include the formally single U=C bond in [U{(CH_2_SiMe*t*Bu)NC_2_H_4_)(N(SiMe_2_*t*Bu)C_2_H_4_)_2_N}] of 2.75(1) Å,[20] the bonds to bridging CH_3_ groups in [Li_3_(Me_2_NC_2_H_4_NMe_2_)_4_(C_5_MeH_4_)][{(η-C_5_MeH_4_)_3_U}_2_CH_3_] of 2.71(3) and 2.74(3) Å,[21] and the formally double U=C bond of 2.779(12) Å in the methanide complex [U(BIPM^Mes^H)(Cl)_3_(thf)] (BIPM^Mes^={C(PPh_2_NMes)_2_}^2−^; Mes=C_6_H_2_-2,4,6-Me_3_).[22] Other examples include dative interactions in a variety of N-heterocyclic carbene and carborane complexes such as [U(C{NMeCMe}_2_)N′′_3_], which has a dative U=C bond length of 2.672(5) Å.[23]

To probe further the effects of pressure on uranium–CH interactions, we have studied **1** using the QTAIM.[24] This approach, focusing on the topology of the electron density *ρ*, is appealing as it has a clear definition of a chemical bond. In the QTAIM, a chemical bond is evidenced by the existence of a bond path (BP) between two atoms, that is, a line of locally maximal electron density, the minimum point on which is a stationary point in the electron density known as the bond critical point (BCP). We have successfully used BCPs to characterize and quantify chemical bonding type[25] and strength,[25e,^26^] and others have applied the approach to agostic bonding.[12a,b]

As the hydrogen atoms were not located experimentally, we optimized the hydrogen atom positions at the heavy atom coordinates from the crystal structures of **1** at the six measured pressures (see the Supporting Information). In all cases the ground electronic state calculated was ^5^A_g_ (*C_i_* point group; confirmed by test calculations at 0 and 3.2 GPa), and the natural spin densities for the U atoms were found to be 2.120 and 2.076 au, respectively, at ambient and highest pressure. QTAIM calculations were performed on the electron densities obtained at each of the six geometries obtained.

Tognetti et al. have employed the QTAIM to study agostic bonding in a series of first-row transition-metal (Ti–Ni) organometallic complexes.[12b] Following a geometric definition, they state that “a H atom (on a carbon) will be defined as ”agostic“ if the corresponding C=H bond length is greater than or equal to 1.101 Å” and “then…=.an agostic bond (at a stationary geometry) will be said to exist if and only if there exists a BP between the metal and a given H atom.” Although the present QTAIM calculations have not been performed on stationary structures (only the H positions have been optimized), we have looked for BPs (or, more correctly for non-equilibrium structures, atomic interaction lines) between the U atom and a H atom attached to C10 (Figure 2). The approach of this C atom to the U center upon compression is accompanied by a pronounced decrease in the short U=C10H atom distance from 2.855 Å at 0 GPa to 2.465 Å at 3.2 GPa. QTAIM analysis finds that while there is no U=H atomic interaction line in the structures between ambient and 2.3 GPa, one appears at 3.2 GPa, suggesting a pressure-induced U=H=C agostic interaction in **1**. Figure 3 a shows the QTAIM molecular graph (MG) of **1** at 0 GPa. Figure 3 b shows the analogous MG at 3.2 GPa. A new atomic interaction line and BCP can be seen between the U center and an H atom on the C10 atom.[27]

**Figure 3:**
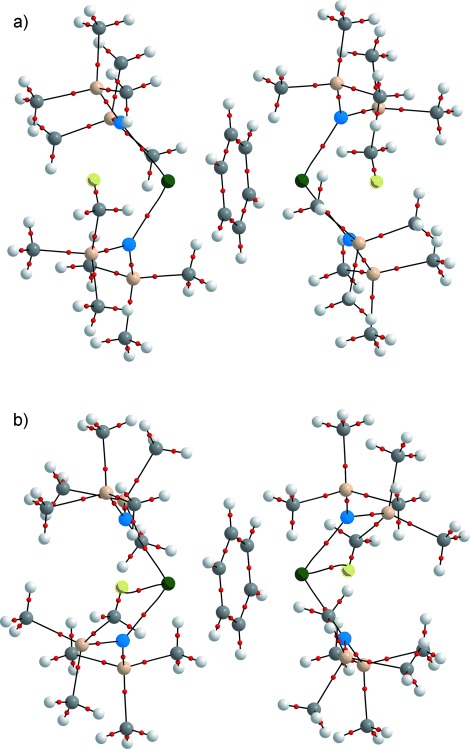
QTAIM molecular graphs of 1 at a) ambient and b) 3.2 GPa with the H of the U=H agostic interaction shown in yellow. Atomic interaction lines are black, bond critical points red. Uranium–benzene lines omitted for clarity. See Figure 1 for atom labelling. Atom colors: green=U; cream=Si; blue=N; gray=C; H=white.

Selected properties of the BCPs (electron density *ρ*, ∇^2^*ρ*, and energy density *H*) at the minima of the U=H atomic interaction lines and the U=H delocalization indices (DIs; *δ*(U,H)) at 3.2 GPa are collected in Table 2 alongside previously proposed[12b] metal-agostic BCP metric ranges. The latter are small in an absolute sense reflecting the weakness of the agostic interaction. The data for U=H lie at the lower end but well within the ranges of *ρ* and ∇^2^*ρ* values. In a very recent study of Ni^II^ complexes, Scherer et al. have quantified weak and strong agostic interactions as having BCP *ρ* values of 0.015 and 0.082 au, respectively.[12c] Local quantities such as BCP *ρ*, ∇^2^*ρ*, and *H* are but part of a QTAIM analysis, and integrated properties such as DIs are “probably more adapted for the description of such a chemical problem”.[12b] Herein, if two atoms are connected by an atomic interaction line the DI can be taken as a measure of bond order. These DI data reveal a small U=H bond order in **1** at the highest pressure, reinforcing the assignment of an agostic bond.

**Table 2:** U=H distance and QTAIM metrics for 1 at 3.2 GPa.

	*r*(U=H)[b]	*ρ*[c]	∇^2^*ρ*[c]	*H*[c]	*δ*(U,H)[c]
**1**	2.455	0.029	0.084	−0.0008	0.082
Literature[a]		0.01–0.13	0.03–0.25		

[a] Range of data from Ref. [12b].

[b] Measured in Å.

[c] Measured in atomic units (au).

Although the QTAIM provides good evidence for an agostic bond in **1** at high pressure, we recognize that the use of QTAIM BPs and BCPs to quantify chemical bonding has been the subject of debate.[12c] We therefore sought additional, independent evidence from orbital structure analysis. The canonical molecular orbitals in large, low symmetry systems are typically highly delocalized and so we focused on localized orbitals using NBO analysis. Figure 4 shows the α spin natural localized molecular orbital (NLMO) that contains most C10=H bonding character in the structures at 0 and 3.2 GPa. The change in composition of this NLMO to include a small but clear U contribution is seen at 3.2 GPa. Furthermore, the U=H Wiberg bond index rises from 0.038 at ambient pressure to 0.073 at 3.2 GPa. These NLMO and bond index data reinforce the conclusion of an increased U=H interaction at high pressure.

**Figure 4:**
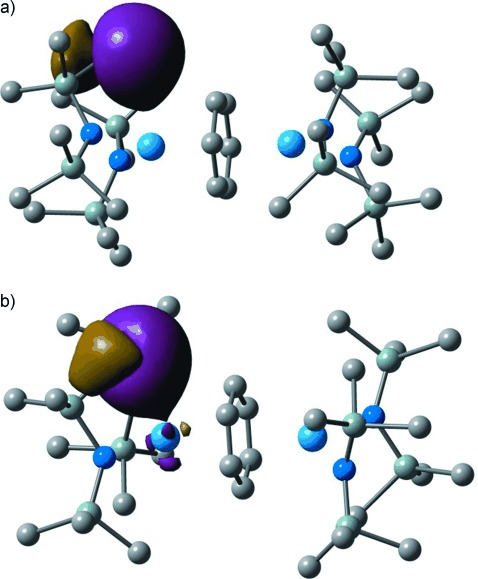
The α spin NLMO (number 156) of 1 at a) 0 GPa and b) 3.2 GPa. NLMO compositions: 0 GPa; 60.2 % C, 35.5 % H, 2.6 % U. 3.2 GPa; 57.2 % C, 37.5 % H, 4.5 % U. H atoms omitted for clarity. Wavefunction cutoff=0.023. Atom colors: blue=U; dark blue=N; blue/gray=Si; gray=C.

To conclude, the dinuclear uranium complex [UN′′_2_]_2_(μ-C_6_H_6_) (**1**) has a short U⋅⋅U separation and close metal-to-hydrocarbyl C=H contacts at ambient pressure that should not be described as agostic.[1a] Pressurizing crystals of **1** dramatically decreases the unit-cell volume by 15 %. Contacts between molecules shorten as the space is decreased.

Application of pressure shortens some U⋅⋅⋅CH contacts in **1**, and the evolution of an agostic interaction between the metal center and C=H bond is computationally characterized for the first time in an organoactinide complex using the QTAIM and NBO methods. The lack of a U=H atomic interaction line at ambient pressure confirms that the U=C10H interaction is not agostic, but the emergence of such a line, and associated BCP, at 3.2 GPa is good evidence for agosticity. The values of the U=H BCP metrics lie within the ranges previously identified for agostic bonds in transition-metal organometallics, and the U=H DI is indicative of a small U=H bond order. NBO analysis reveals an approximate doubling of the (admittedly small) U=H Wiberg bond index over the pressure range 0–3.2 GPa, and a similar increase is observed in the U contribution to the C10=H NLMO.

Taken together, the present QTAIM and NBO data provide evidence of an enhanced U=H interaction at high pressure. This interaction may be characterized in the QTAIM sense as moving from non-agostic to agostic. However, whether the application of pressure induces a fundamental change in bonding type is open to debate. As many coordination and organometallic crystals can be subjected to much higher pressures than reported herein (circa 6 GPa) without loss of diffraction as happens for **1**, the present bonding analysis provides a conservative description of possible changes in bonding in these systems that can be brought about by the application of pressure. Reactions, such as polymerization, that occur in the solid state are well-documented, but are usually initiated by heat or light.[28] Work is in progress to identify other pressure-induced reactions that might be possible within the crystal.
