# Supplementary material for: Characterizing Pressure-Induced Uranium C=H Agostic Bonds
Source: Angew Chem Int Ed Engl. 2015 Apr 16;54(23):6735–9. doi: 10.1002/anie.201411250 (PMC4515102; doi:10.1002/anie.201411250)
Supplement: Supplementary file 2 [file anie0054-6735-sd2.pdf]

Angewandte Chemie
Eine Zeitschrift der Gesellschaft Deutscher Chemiker

Supporting Information

German Edition: DOI:

Characterizing Pressure-Induced Uranium C–H Agostic Bonds**

Polly L. Arnold, Alessandro Prescimone, Joy H. Farnaby, Stephen M. Mansell, Simon Parsons,* and Nikolas Kaltsoyannis**

anie_201411250_sm_miscellaneous_information.pdf
anie_201411250_sm_compression_of_1.mpg

Table of Contents

Synthetic details 1

Crystallographic details 1

General details 1

Crystallographic details for the pressure study 2

Details for the animation of the compression of [UN"$_2$]$_2$(μ-C_6H_6) 1 2

Quantum chemical calculations 4

Computational coordinates 4

References 10

Synthetic details

[N"$_2$U]$_2$(μ-C_6H_6) **1** was synthesized as previously described (N" = N(SiMe$_3$)$_2$.[1] All crystal manipulations were carried out under a dry, oxygen-free dinitrogen atmosphere using standard Schlenk techniques and coated in Fluorinert F70 oil before being transferred rapidly to the pressure cell.

Crystallographic details

CIF data are deposited with the CCDC, codes 1032195-9.

General details

Atmospheric pressure X-ray diffraction data were collected on an Oxford Diffraction Supernova diffractometer using mirror monochromated Cu-Kα radiation (λ = 1.54178 Å) at 100K for [(ODtbp)$_2$U]$_2$(μ-C_6H_6), or on the same diffractometer with Mo-*K*α radiation (λ = 0.71073 Å) at 120K for **1**. Compound **1** crystallized solvent-free in the solid-state (space group $P2_1/c$).

High-pressure single crystal experiments were carried out using a Merrill-Bassett diamond anvil cell (half-opening angle 40°),[2] equipped with Boehler-Almax diamonds with 600 μm culets and a tungsten gasket.[3] Fluorinert F70 was used as hydrostatic medium and a small ruby chip was loaded into the cell as the pressure calibrant with the ruby fluorescence used to measure the pressure.[4] Data collections were taken at ambient pressure, 0.24, 0.45, 1.15, 2.20, 3.35, 4.8 and 6.0 GPa for **1**. Starting models for refinement of the high-pressure structures were taken from the ambient-pressure results. The program CRYSTALS[9] was used to refine the structures of **1** against *F* using the reflections with $I > 2\sigma(I)$. Due to the low completeness of the data sets (a result of shading of reciprocal space by the pressure cell), thermal and vibrational similarity restraints were used for all the non-H and non-U atoms. All the H atoms were placed geometrically, and all the aromatic rings in the aryloxide and hydrazonido ligands for compound **1** refined as rigid groups. Compound **1** becomes twinned after the phase transition and the consequent symmetry loss meant all the C atoms in the new phase had to be refined isotropically. Additional details are reported in Table SI1.

The peripheral X ligands in **1** adopt an eclipsed geometry. This is in contrast to the bis aryloxide complexes, [U(ODtbp)$_2$]$_2$(C_6H_5R) (R = H, Me) for which a variety of structures that

differ in the identity of the central arene and the presence and identity of lattice solvent have been collected and show a range in the angle between the two UX_2 planes from 53.5° to 89.49°. This suggests that relatively subtle changes in crystal packing can influence the geometry at the metal.

Crystallographic details for the pressure study

Table SI1 crystallographic details on the pressure study of **1**

Compound reference	**1** 0.0GPa	**1** 0.8GPa	**1** 1.3GPa	**1** 1.8GPa	**1** 2.3GPa	**1** 3.2GPa
Chemical formula	$C_{30}H_{78}N_4Si_8U_2$					
Formula Mass	1195.72	1195.72	1195.72	2391.44	2391.44	2391.44
Crystal system	Monoclinic	Monoclinic	Monoclinic	Triclinic	Triclinic	Triclinic
a/Å	10.3378(3)	10.2191(8)	10.1083(6)	10.0303(6)	10.0196(7)	9.9474(9)
b/Å	17.6019(5)	17.2201(15)	17.0028(12)	16.6033(18)	16.5632(18)	16.375(3)
c/Å	13.7838(5)	13.6566(12)	13.5677(9)	13.3760(12)	13.3399(13)	13.2103(18)
α/°	90	90	90	91.289(9)	91.361(9)	91.439(13)
β/°	107.967(4)	109.048(6)	109.290(3)	109.501(5)	109.687(5)	110.007(7)
γ/°	90	90	90	89.763(6)	89.954(6)	89.909(9)
Unit cell volume/$Å^3$	2385.86(14)	2271.6(3)	2201.0(3)	2099.3(3)	2083.8(3)	2021.3(5)
Temperature/K	100	300	300	300	300	300
Space group	$P2_1/c$	$P2_1/c$	$P2_1/c$	$P\bar{1}$	$P\bar{1}$	$P\bar{1}$
No. of formula units per unit cell, Z	2	2	2	1	1	1
Radiation type	Mo Kα	Mo Kα	Mo Kα	Mo Kα	Mo Kα	Mo Kα
Absorption coefficient, μ/mm^{-1}	7.004	7.356	7.592	7.960	8.019	8.267
No. of reflections measured	33307	8630	7556	7368	6021	6846
No. of independent reflections	4868	2332	2175	2280	2302	2067
R_{int}	0.041	0.038	0.047	0.056	0.055	0.079
Final R_1 values ($I > 2\sigma(I)$)	0.0201	0.0624	0.1243	0.0678	0.0743	0.0704
Final $wR(F^2)$ values ($I > 2\sigma(I)$)	0.0164	0.0099	0.0936	0.0496	0.0567	0.0681
Final R_1 values (all data)	0.0248	0.0909	0.1436	0.0993	0.1096	0.1088
Final $wR(F^2)$ values (all data)	0.0174	0.0112	0.1076	0.0602	0.1227	0.0903

Details for the animation of the compression of $[UN''_2]_2(\mu\text{-}C_6H_6)$ 1

The movie associated with the SI shows the compression series for **1**. C4 and C10 are highlighted in orange. The first effect of the phase change is to collapse interstitial voids. C4 and 10 face each other across the void, and the N" ligands, which were quite distant, are forced to 'butt' into each other. In particular, the methyl groups C5 and C12, which are positioned on the upper surface of the plots, are pushed right into each other. C4 and C10, which are part of the same $SiMe_3$ groups are pushed closer to the U centres in order to accommodate this strain.

The data are insufficiently precise to identify whether the C-C bond lengths in the arene in **1** are lengthened by any degree.

Table SI2. Comparison of selected metrics (U···U separation, and key close U···CH contacts) at ambient and high pressure for 1.

		**distance (Å)**		
**complex**	Atom pair	Ambient pressure	3.2 GPa	comment
**1**	U(1)-U(1')	4.2492(2)	4.183(6)	0.066 Å contraction
	U(1)-C(10)	3.022(3)	2.95(2)	closest silylamide C-H agostic

Table SI3 The U···U separation as a function of pressure for **1**. Distances in Å

Pressure (GPa)	**1**
0	4.2492(2)
0.8	4.2367(8)
1.3	4.226(2)
1.8	4.206(4)
2.3	4.202(5)
3.2	4.191(5)

Table SI4 Direct comparison of the shortest U···C separation as a function of pressure for **1**. Distances in Å

**Compound**	**1**
shortest U···C separation<br>Pressure (GPa)	U···C(10)
0	3.022(3)
0.8	3.038(12)
1.3	3.00(3)
1.8	3.00(2)
2.3	2.98(2)
3.2	2.96(2)

Quantum chemical calculations

Spin-unrestricted density functional theory calculations were performed on **1** with the Gaussian 09 code, Revision C.01.[10] A (14s 13p 10d 8f 6g)/[10s 9p 5d 4f 3g] segmented valence basis set with Stuttgart-Bonn variety relativistic pseudopotential was used for uranium,[11] and the cc-pVTZ basis sets of Dunning for the other elements. The PBE functional was employed,[12] in conjunction with the ultrafine integration grid and the standard SCF convergence criterion (10^{-8}). For each of the structures at the six different pressures (ambient, 0.8, 1.3, 1.8, 2.3 and 3.2 GPa), the positions of the heavy atoms were fixed to those obtained experimentally, and those of the H atoms were optimized using the standard geometry convergence criteria. QTAIM analyses were performed using the AIMALL program package,[13] with .wfx files generated in Gaussian used as input. Natural bond orbital analyses were performed using the GenNBO6 code,[14] using .47 files from G09 as input.

Computational coordinates

Converged Cartesian coordinates for **1** at ambient pressure.
SCF energy = -4677.0578015 H

U	0.893727	1.164577	-1.535909
N	-0.277156	2.090930	-3.308718
N	3.118682	1.729563	-1.854639
Si	-0.365946	3.785641	-3.047666
Si	-0.967676	1.334928	-4.695273
Si	4.087449	2.811728	-0.922534
Si	3.686482	0.893120	-3.248536
C	0.249313	1.068787	0.955837
C	1.000808	-1.032351	-0.197986
C	1.245310	0.039428	0.740807
C	0.485597	4.158801	-1.408189
C	-2.071412	4.509607	-2.841289
C	0.544582	4.747232	-4.363549
C	-2.592646	2.108708	-5.209248
C	-1.313930	-0.486693	-4.412062
C	0.152951	1.478560	-6.193931
C	2.361006	-0.384425	-3.675188
C	3.890604	1.997640	-4.745097
C	5.246717	-0.112652	-3.003873
C	3.294021	3.152324	0.744740
C	5.795238	2.149192	-0.519220
C	4.349168	4.445536	-1.817271
H	0.324696	5.225754	-1.191822
H	0.063183	3.628089	-0.532973
H	1.575734	4.022426	-1.421910
H	-1.988175	5.552437	-2.497480
H	-2.655284	3.959864	-2.088085
H	-2.657095	4.517829	-3.769073
H	0.575000	5.824340	-4.136661
H	1.582014	4.398512	-4.464044
H	0.063014	4.640314	-5.346659
H	-3.039272	1.519778	-6.025733
H	-2.466613	3.133306	-5.587190
H	-3.323587	2.140720	-4.388979
H	-1.679359	-0.928867	-5.352201
H	-2.099159	-0.635084	-3.656968
H	-0.440249	-1.074228	-4.100771
H	-0.321770	1.013389	-7.072243
H	0.344743	2.531212	-6.446846
H	1.130871	0.996162	-6.061044
H	2.761999	-1.039379	-4.463585
H	2.103871	-1.058579	-2.837470
H	1.436718	0.032407	-4.100380
H	4.138437	1.418851	-5.648221
H	4.697452	2.730575	-4.598202
H	2.967484	2.556935	-4.948882
H	5.399234	-0.773693	-3.871202
H	6.146331	0.508739	-2.906571
H	5.182857	-0.746595	-2.107968
H	3.902032	3.896351	1.283127
H	2.271892	3.550641	0.700293
H	3.272464	2.243857	1.363464
H	6.272751	2.802486	0.227679
H	5.754736	1.136407	-0.093644
H	6.460709	2.118827	-1.392588
H	4.931385	5.142681	-1.194701
H	3.411976	4.951865	-2.087079
H	4.913252	4.294261	-2.749454
H	2.172487	0.053750	1.307629
H	0.432632	1.867203	1.672034
H	1.751486	-1.806986	-0.345326
C	-0.249313	-1.068787	-0.955837
C	-1.000808	1.032351	0.197986
C	-1.245310	-0.039428	-0.740807
H	-2.172487	-0.053750	-1.307629
H	-0.432632	-1.867203	-1.672034
H	-1.751486	1.806986	0.345326
U	-0.893727	-1.164577	1.535909
N	0.277156	-2.090930	3.308718
N	-3.118682	-1.729563	1.854639
Si	0.365946	-3.785641	3.047666
Si	0.967676	-1.334928	4.695273
Si	-4.087449	-2.811728	0.922534
Si	-3.686482	-0.893120	3.248536
C	-0.485597	-4.158801	1.408189
C	2.071412	-4.509607	2.841289
C	-0.544582	-4.747232	4.363549
C	2.592646	-2.108708	5.209248
C	1.313930	0.486693	4.412062
C	-0.152951	-1.478560	6.193931
C	-2.361006	0.384425	3.675188
C	-3.890604	-1.997640	4.745097
C	-5.246717	0.112652	3.003873
C	-3.294021	-3.152324	-0.744740
C	-5.795238	-2.149192	0.519220
C	-4.349168	-4.445536	1.817271
H	-0.324696	-5.225754	1.191822
H	-0.063183	-3.628089	0.532973
H	-1.575734	-4.022426	1.421910
H	1.988175	-5.552437	2.497480
H	2.655284	-3.959864	2.088085
H	2.657095	-4.517829	3.769073
H	-0.575000	-5.824340	4.136661
H	-1.582014	-4.398512	4.464044

H -0.063014 -4.640314 5.346659
H 3.039272 -1.519778 6.025733
H 2.466613 -3.133306 5.587190
H 3.323587 -2.140720 4.388979
H 1.679359 0.928867 5.352201
H 2.099159 0.635084 3.656968
H 0.440249 1.074228 4.100771
H 0.321770 -1.013389 7.072243
H -0.344743 -2.531212 6.446846
H -1.130871 -0.996162 6.061044
H -2.761999 1.039379 4.463585
H -2.103871 1.058579 2.837470
H -1.436718 -0.032407 4.100380
H -4.138437 -1.418851 5.648221
H -4.697452 -2.730575 4.598202
H -2.967484 -2.556935 4.948882
H -5.399234 0.773693 3.871202
H -6.146331 -0.508739 2.906571
H -5.182857 0.746595 2.107968
H -3.902032 -3.896351 -1.283127
H -2.271892 -3.550641 -0.700293
H -3.272464 -2.243857 -1.363464
H -6.272751 -2.802486 -0.227679
H -5.754736 -1.136407 0.093644
H -6.460709 -2.118827 1.392588
H -4.931385 -5.142681 1.194701
H -3.411976 -4.951865 2.087079
H -4.913252 -4.294261 2.749454

Converged Cartesian coordinates for **1** at 0.8 GPa.
SCF energy = -4677.041368 H

U -0.929949 1.142898 1.521952
Si 0.325244 3.729185 3.047777
Si 0.895573 1.263955 4.687199
Si -4.100001 2.808598 0.917818
Si -3.708796 0.854117 3.238828
N 0.212839 2.035416 3.290463
N -3.155909 1.703068 1.844673
C -0.210046 1.076256 -0.930728
C 0.982769 1.045260 -0.167815
C 1.188988 -0.058548 0.768076
C -0.486418 4.131102 1.435463
C 2.085403 4.420400 2.854144
C -0.574137 4.676979 4.379969
C 2.540381 1.942427 5.203553
C 1.179082 -0.528657 4.443222
C -0.174811 1.434434 6.174298
C -2.387598 -0.459777 3.650620
C -3.931071 1.973423 4.714308
C -5.299122 -0.123985 2.961288
C -3.266780 3.125448 -0.702241
C -5.789294 2.181787 0.491827
C -4.333181 4.406624 1.874363
H -0.385842 1.876504 -1.649404
H 1.749974 1.810554 -0.270702
H 2.100413 -0.077273 1.360421
H -0.308160 5.197461 1.228461
H -1.578983 4.014092 1.431642
H -0.063361 3.604754 0.558070
H 2.034513 5.472167 2.532909
H 2.669019 4.384981 3.782261
H 2.643149 3.862754 2.087422
H -0.600722 5.757874 4.171035
H -0.092745 4.552415 5.360732
H -1.612823 4.330336 4.474719
H 2.942760 1.321743 6.019571
H 2.478923 2.970608 5.588573
H 3.284036 1.937768 4.393501
H 1.541625 -0.953480 5.393042
H 0.301263 -1.119925 4.152807
H 1.960451 -0.724194 3.693556
H 0.294220 0.931258 7.034891
H -0.321881 2.485885 6.461121
H -1.176819 0.997675 6.057537
H -2.824174 -1.149485 4.388676
H -1.482262 -0.061417 4.125208
H -2.104960 -1.092162 2.789030
H -4.188158 1.414633 5.627594
H -3.016654 2.546039 4.920026
H -4.742174 2.694470 4.537323
H -5.479277 -0.803058 3.809151
H -6.178973 0.526072 2.871012
H -5.239443 -0.734465 2.049177
H -3.816857 3.901547 -1.258428
H -2.225294 3.466906 -0.640502
H -3.279918 2.218879 -1.324763
H -6.261913 2.851284 -0.244452
H -6.463150 2.135500 1.358123
H -5.755270 1.177831 0.044591
H -4.852165 5.168945 1.271964
H -4.950149 4.228509 2.767363
H -3.389703 4.850192 2.220869
C 0.210046 -1.076256 0.930728
C -0.982769 -1.045260 0.167815
C -1.188988 0.058548 -0.768076
H 0.385842 -1.876504 1.649404
H -1.749974 -1.810554 0.270702
H -2.100413 0.077273 -1.360421
U 0.929949 -1.142898 -1.521952
Si -0.325244 -3.729185 -3.047777
Si -0.895573 -1.263955 -4.687199
Si 4.100001 -2.808598 -0.917818
Si 3.708796 -0.854117 -3.238828
N -0.212839 -2.035416 -3.290463
N 3.155909 -1.703068 -1.844673
C 0.486418 -4.131102 -1.435463
C -2.085403 -4.420400 -2.854144
C 0.574137 -4.676979 -4.379969
C -2.540381 -1.942427 -5.203553
C -1.179082 0.528657 -4.443222
C 0.174811 -1.434434 -6.174298
C 2.387598 0.459777 -3.650620
C 3.931071 -1.973423 -4.714308
C 5.299122 0.123985 -2.961288
C 3.266780 -3.125448 0.702241
C 5.789294 -2.181787 -0.491827
C 4.333181 -4.406624 -1.874363
H 0.308160 -5.197461 -1.228461
H 1.578983 -4.014092 -1.431642
H 0.063361 -3.604754 -0.558070
H -2.034513 -5.472167 -2.532909
H -2.669019 -4.384981 -3.782261
H -2.643149 -3.862754 -2.087422
H 0.600722 -5.757874 -4.171035
H 0.092745 -4.552415 -5.360732
H 1.612823 -4.330336 -4.474719
H -2.942760 -1.321743 -6.019571
H -2.478923 -2.970608 -5.588573
H -3.284036 -1.937768 -4.393501
H -1.541625 0.953480 -5.393042
H -0.301263 1.119925 -4.152807
H -1.960451 0.724194 -3.693556
H -0.294220 -0.931258 -7.034891
H 0.321881 -2.485885 -6.461121
H 1.176819 -0.997675 -6.057537
H 2.824174 1.149485 -4.388676
H 1.482262 0.061417 -4.125208

H 2.104960 1.092162 -2.789030
H 4.188158 -1.414633 -5.627594
H 3.016654 -2.546039 -4.920026
H 4.742174 -2.694470 -4.537323
H 5.479277 0.803058 -3.809151
H 6.178973 -0.526072 -2.871012
H 5.239443 0.734465 -2.049177
H 3.816857 -3.901547 1.258428
H 2.225294 -3.466906 0.640502
H 3.279918 -2.218879 1.324763
H 6.261913 -2.851284 0.244452
H 6.463150 -2.135500 -1.358123
H 5.755270 -1.177831 -0.044591
H 4.852165 -5.168945 -1.271964
H 4.950149 -4.228509 -2.767363
H 3.389703 -4.850192 -2.220869

Converged Cartesian coordinates for **1** at 1.3 GPa.
SCF energy = -4677.0395775 H

U -0.950920 1.131876 1.509699
Si 0.297780 3.698109 3.056790
Si 0.869105 1.178294 4.679309
Si -4.114581 2.815664 0.893858
Si -3.690022 0.822936 3.236074
N 0.187779 1.974025 3.275773
N -3.171846 1.718983 1.813329
C -0.242953 1.103482 -0.980939
C 0.978571 1.030370 -0.167758
C 1.208031 -0.064611 0.793972
C -0.512101 4.073871 1.434271
C 2.035274 4.461535 2.906960
C -0.630946 4.573753 4.430873
C 2.509964 1.870308 5.160815
C 1.141345 -0.615501 4.392455
C -0.218427 1.377227 6.198100
C -2.350018 -0.493081 3.624096
C -3.945135 2.040336 4.686993
C -5.281991 -0.170028 2.932572
C -3.307124 3.179524 -0.717135
C -5.782714 2.159356 0.435404
C -4.376527 4.403725 1.818451
H -0.397494 1.915692 -1.686735
H 1.741134 1.803036 -0.257024
H 2.127733 -0.042822 1.373408
H -0.365566 5.142767 1.215869
H -1.601253 3.926030 1.431328
H -0.068743 3.549004 0.566111
H 1.935192 5.516208 2.607764
H 2.599944 4.433691 3.846949
H 2.634138 3.950096 2.139401
H -0.685253 5.662104 4.270615
H -0.137049 4.417548 5.400329
H -1.658884 4.197221 4.514153
H 2.949720 1.260193 5.965684
H 2.440032 2.897612 5.546096
H 3.228695 1.878203 4.328548
H 1.517040 -1.063533 5.326861
H 0.261610 -1.198349 4.096681
H 1.915479 -0.784424 3.629769
H 0.224894 0.836744 7.049444
H -0.304732 2.432222 6.492922
H -1.240587 0.995677 6.068577
H -2.819203 -1.228176 4.295329
H -1.476475 -0.109496 4.160265
H -2.025671 -1.073100 2.740080
H -4.219209 1.541030 5.628686
H -3.033561 2.624254 4.863641
H -4.754826 2.737477 4.431416
H -5.481138 -0.850679 3.774635
H -6.156666 0.482837 2.820002
H -5.193056 -0.775979 2.020007
H -3.921564 3.907414 -1.271242
H -2.296600 3.605643 -0.662337
H -3.247763 2.275716 -1.341342
H -6.265708 2.830948 -0.292024
H -6.467182 2.073295 1.290804
H -5.715883 1.166344 -0.032776
H -4.894863 5.149949 1.195335
H -5.000578 4.245250 2.710263
H -3.441022 4.865672 2.164796
C 0.242953 -1.103482 0.980939
C -0.978571 -1.030370 0.167758
C -1.208031 0.064611 -0.793972
H 0.397494 -1.915692 1.686735
H -1.741134 -1.803036 0.257024
H -2.127733 0.042822 -1.373408
U 0.950920 -1.131876 -1.509699
Si -0.297780 -3.698109 -3.056790
Si -0.869105 -1.178294 -4.679309
Si 4.114581 -2.815664 -0.893858
Si 3.690022 -0.822936 -3.236074
N -0.187779 -1.974025 -3.275773
N 3.171846 -1.718983 -1.813329
C 0.512101 -4.073871 -1.434271
C -2.035274 -4.461535 -2.906960
C 0.630946 -4.573753 -4.430873
C -2.509964 -1.870308 -5.160815
C -1.141345 0.615501 -4.392455
C 0.218427 -1.377227 -6.198100
C 2.350018 0.493081 -3.624096
C 3.945135 -2.040336 -4.686993
C 5.281991 0.170028 -2.932572
C 3.307124 -3.179524 0.717135
C 5.782714 -2.159356 -0.435404
C 4.376527 -4.403725 -1.818451
H 0.365566 -5.142767 -1.215869
H 1.601253 -3.926030 -1.431328
H 0.068743 -3.549004 -0.566111
H -1.935192 -5.516208 -2.607764
H -2.599944 -4.433691 -3.846949
H -2.634138 -3.950096 -2.139401
H 0.685253 -5.662104 -4.270615
H 0.137049 -4.417548 -5.400329
H 1.658884 -4.197221 -4.514153
H -2.949720 -1.260193 -5.965684
H -2.440032 -2.897612 -5.546096
H -3.228695 -1.878203 -4.328548
H -1.517040 1.063533 -5.326861
H -0.261610 1.198349 -4.096681
H -1.915479 0.784424 -3.629769
H -0.224894 -0.836744 -7.049444
H 0.304732 -2.432222 -6.492922
H 1.240587 -0.995677 -6.068577
H 2.819203 1.228176 -4.295329
H 1.476475 0.109496 -4.160265
H 2.025671 1.073100 -2.740080
H 4.219209 -1.541030 -5.628686
H 3.033561 -2.624254 -4.863641
H 4.754826 -2.737477 -4.431416
H 5.481138 0.850679 -3.774635
H 6.156666 -0.482837 -2.820002
H 5.193056 0.775979 -2.020007
H 3.921564 -3.907414 1.271242
H 2.296600 -3.605643 0.662337
H 3.247763 -2.275716 1.341342
H 6.265708 -2.830948 0.292024
H 6.467182 -2.073295 -1.290804

H 5.715883 -1.166344 0.032776
H 4.894863 -5.149949 -1.195335
H 5.000578 -4.245250 -2.710263
H 3.441022 -4.865672 -2.164796

Converged Cartesian coordinates for **1** at 1.8 GPa.
SCF energy = -4677.0024471 H

U -1.031809 1.096288 1.468040
Si 0.402940 3.467254 3.015243
Si 0.723701 1.024515 4.664046
Si -4.105483 2.682784 0.753810
Si -3.826266 0.851539 3.195502
N 0.114908 1.869137 3.277438
N -3.244580 1.637096 1.777379
C -0.183542 0.958114 -1.042478
C 0.950778 0.983003 -0.152527
C -1.166918 -0.063415 -0.874824
C -0.543389 4.066805 1.525269
C 2.147167 3.989883 2.735400
C -0.252780 4.615598 4.449752
C 2.272547 1.739520 5.357350
C 1.004379 -0.778169 4.348908
C -0.504107 1.174105 6.138893
C -2.526311 -0.624443 3.416098
C -3.840114 1.987498 4.664046
C -5.429409 -0.115334 2.924483
C -3.118694 3.040697 -0.844571
C -5.738909 2.070311 0.226899
C -4.421195 4.228601 1.714352
H -0.316669 1.770834 -1.752407
H 1.658460 1.805707 -0.278410
H -2.051704 -0.118488 -1.501969
H -0.310224 5.138136 1.430959
H -1.636304 4.006457 1.621058
H -0.245200 3.631343 0.551850
H 2.178094 5.055702 2.461099
H 2.773931 3.870071 3.628547
H 2.632080 3.429701 1.920685
H -0.096172 5.675888 4.200230
H 0.270046 4.418643 5.396837
H -1.327940 4.463631 4.623390
H 2.567057 1.158777 6.245435
H 2.131987 2.777867 5.689267
H 3.126756 1.725269 4.664643
H 1.373965 -1.229219 5.284565
H 1.778735 -0.956005 3.589149
H 0.116663 -1.350314 4.058528
H -0.069149 0.704481 7.034557
H -0.695078 2.230523 6.374582
H -1.473370 0.694155 5.950015
H -2.858565 -1.259346 4.251165
H -1.497805 -0.344853 3.683868
H -2.518774 -1.256840 2.515172
H -4.048050 1.469466 5.612105
H -2.866068 2.484872 4.756893
H -4.604594 2.768679 4.542015
H -5.604289 -0.811936 3.758436
H -6.318130 0.524610 2.838722
H -5.346881 -0.704187 2.000730
H -3.653800 3.815708 -1.416061
H -2.097729 3.411094 -0.690637
H -3.065902 2.145078 -1.478583
H -6.195067 2.766437 -0.494994
H -6.441240 1.980051 1.067283
H -5.690296 1.086507 -0.263429
H -4.899600 5.023753 1.119769
H -5.108323 4.003867 2.544924
H -3.517519 4.662601 2.163653
C 0.183542 -0.958114 1.042478
C -0.950778 -0.983003 0.152527
C 1.166918 0.063415 0.874824
H 0.316669 -1.770834 1.752407
H -1.658460 -1.805707 0.278410
H 2.051704 0.118488 1.501969
U 1.031809 -1.096288 -1.468040
Si -0.402940 -3.467254 -3.015243
Si -0.723701 -1.024515 -4.664046
Si 4.105483 -2.682784 -0.753810
Si 3.826266 -0.851539 -3.195502
N -0.114908 -1.869137 -3.277438
N 3.244580 -1.637096 -1.777379
C 0.543389 -4.066805 -1.525269
C -2.147167 -3.989883 -2.735400
C 0.252780 -4.615598 -4.449752
C -2.272547 -1.739520 -5.357350
C -1.004379 0.778169 -4.348908
C 0.504107 -1.174105 -6.138893
C 2.526311 0.624443 -3.416098
C 3.840114 -1.987498 -4.664046
C 5.429409 0.115334 -2.924483
C 3.118694 -3.040697 0.844571
C 5.738909 -2.070311 -0.226899
C 4.421195 -4.228601 -1.714352
H 0.310224 -5.138136 -1.430959
H 1.636304 -4.006457 -1.621058
H 0.245200 -3.631343 -0.551850
H -2.178094 -5.055702 -2.461099
H -2.773931 -3.870071 -3.628547
H -2.632080 -3.429701 -1.920685
H 0.096172 -5.675888 -4.200230
H -0.270046 -4.418643 -5.396837
H 1.327940 -4.463631 -4.623390
H -2.567057 -1.158777 -6.245435
H -2.131987 -2.777867 -5.689267
H -3.126756 -1.725269 -4.664643
H -1.373965 1.229219 -5.284565
H -1.778735 0.956005 -3.589149
H -0.116663 1.350314 -4.058528
H 0.069149 -0.704481 -7.034557
H 0.695078 -2.230523 -6.374582
H 1.473370 -0.694155 -5.950015
H 2.858565 1.259346 -4.251165
H 1.497805 0.344853 -3.683868
H 2.518774 1.256840 -2.515172
H 4.048050 -1.469466 -5.612105
H 2.866068 -2.484872 -4.756893
H 4.604594 -2.768679 -4.542015
H 5.604289 0.811936 -3.758436
H 6.318130 -0.524610 -2.838722
H 5.346881 0.704187 -2.000730
H 3.653800 -3.815708 1.416061
H 2.097729 -3.411094 0.690637
H 3.065902 -2.145078 1.478583
H 6.195067 -2.766437 0.494994
H 6.441240 -1.980051 -1.067283
H 5.690296 -1.086507 0.263429
H 4.899600 -5.023753 -1.119769
H 5.108323 -4.003867 -2.544924
H 3.517519 -4.662601 -2.163653

Converged Cartesian coordinates for **1** at 2.3 GPa.
SCF energy = -4676.9942763 H

U -1.046250 1.092071 1.458281
Si 0.408848 3.464284 3.017264
Si 0.708493 1.008305 4.663386
Si -4.112170 2.669820 0.737051

Si -3.836862 0.841571 3.180495
N 0.112684 1.888952 3.289734
N -3.276994 1.629030 1.757873
C -0.203469 0.969416 -1.081092
C 0.950823 0.988615 -0.190855
C -1.155636 -0.014324 -0.886470
C -0.501492 3.987581 1.494192
C 2.179734 3.973134 2.737260
C -0.285885 4.657122 4.532802
C 2.231712 1.720699 5.386626
C 0.980533 -0.788097 4.369570
C -0.524244 1.172509 6.114887
C -2.547435 -0.613970 3.365072
C -3.874419 1.937312 4.670920
C -5.501650 -0.025800 3.026053
C -3.103148 3.035489 -0.841268
C -5.724457 1.984451 0.125563
C -4.480826 4.247581 1.695092
H -0.331444 1.782989 -1.791131
H 1.670231 1.802124 -0.285160
H -2.046699 -0.106675 -1.501417
H -0.290069 5.055610 1.332549
H -1.597958 3.920793 1.567619
H -0.169345 3.500593 0.555438
H 2.228241 5.037322 2.459793
H 2.804024 3.841679 3.630191
H 2.644821 3.397291 1.921935
H -0.143078 5.720146 4.291597
H 0.257629 4.438134 5.461920
H -1.355766 4.473101 4.701181
H 2.503905 1.148399 6.287239
H 2.077542 2.760896 5.706838
H 3.108070 1.707626 4.721595
H 1.342780 -1.221061 5.316585
H 1.757999 -0.988624 3.618539
H 0.090639 -1.362494 4.088958
H -0.107095 0.701670 7.018659
H -0.701920 2.232703 6.344022
H -1.500656 0.707748 5.923420
H -2.862776 -1.268925 4.191362
H -1.520469 -0.328330 3.638648
H -2.530980 -1.234104 2.455868
H -4.093398 1.380992 5.594841
H -2.921554 2.463338 4.813103
H -4.660428 2.698928 4.556700
H -5.668909 -0.673263 3.900515
H -6.334993 0.689741 2.985963
H -5.553296 -0.652858 2.125379
H -3.638418 3.804594 -1.420627
H -2.086057 3.414807 -0.683278
H -3.041624 2.138663 -1.472730
H -6.183019 2.662943 -0.610693
H -6.455667 1.842071 0.933271
H -5.586728 1.011212 -0.368714
H -4.959392 5.026441 1.081172
H -5.171511 4.019494 2.521182
H -3.579506 4.688509 2.143796
C 0.203469 -0.969416 1.081092
C -0.950823 -0.988615 0.190855
C 1.155636 0.014324 0.886470
H 0.331444 -1.782989 1.791131
H -1.670231 -1.802124 0.285160
H 2.046699 0.106675 1.501417
U 1.046250 -1.092071 -1.458281
Si -0.408848 -3.464284 -3.017264
Si -0.708493 -1.008305 -4.663386
Si 4.112170 -2.669820 -0.737051
Si 3.836862 -0.841571 -3.180495
N -0.112684 -1.888952 -3.289734
N 3.276994 -1.629030 -1.757873
C 0.501492 -3.987581 -1.494192
C -2.179734 -3.973134 -2.737260
C 0.285885 -4.657122 -4.532802
C -2.231712 -1.720699 -5.386626
C -0.980533 0.788097 -4.369570
C 0.524244 -1.172509 -6.114887
C 2.547435 0.613970 -3.365072
C 3.874419 -1.937312 -4.670920
C 5.501650 0.025800 -3.026053
C 3.103148 -3.035489 0.841268
C 5.724457 -1.984451 -0.125563
C 4.480826 -4.247581 -1.695092
H 0.290069 -5.055610 -1.332549
H 1.597958 -3.920793 -1.567619
H 0.169345 -3.500593 -0.555438
H -2.228241 -5.037322 -2.459793
H -2.804024 -3.841679 -3.630191
H -2.644821 -3.397291 -1.921935
H 0.143078 -5.720146 -4.291597
H -0.257629 -4.438134 -5.461920
H 1.355766 -4.473101 -4.701181
H -2.503905 -1.148399 -6.287239
H -2.077542 -2.760896 -5.706838
H -3.108070 -1.707626 -4.721595
H -1.342780 1.221061 -5.316585
H -1.757999 0.988624 -3.618539
H -0.090639 1.362494 -4.088958
H 0.107095 -0.701670 -7.018659
H 0.701920 -2.232703 -6.344022
H 1.500656 -0.707748 -5.923420
H 2.862776 1.268925 -4.191362
H 1.520469 0.328330 -3.638648
H 2.530980 1.234104 -2.455868
H 4.093398 -1.380992 -5.594841
H 2.921554 -2.463338 -4.813103
H 4.660428 -2.698928 -4.556700
H 5.668909 0.673263 -3.900515
H 6.334993 -0.689741 -2.985963
H 5.553296 0.652858 -2.125379
H 3.638418 -3.804594 1.420627
H 2.086057 -3.414807 0.683278
H 3.041624 -2.138663 1.472730
H 6.183019 -2.662943 0.610693
H 6.455667 -1.842071 -0.933271
H 5.586728 -1.011212 0.368714
H 4.959392 -5.026441 -1.081172
H 5.171511 -4.019494 -2.521182
H 3.579506 -4.688509 -2.143796

Converged Cartesian coordinates for **1** at 3.2 GPa.
SCF energy = -4676.9512204 H

U -1.085183 1.085344 1.427015
Si 0.409670 3.415439 2.957023
Si 0.650942 0.995450 4.638419
Si -4.149936 2.716169 0.768106
Si -3.852361 0.814617 3.163009
N 0.072157 1.878663 3.300747
N -3.292475 1.576657 1.700009
C -0.190902 0.977078 -1.044823
C 0.987556 0.915335 -0.249418
C -1.216356 0.058889 -0.811538
C -0.567526 4.036150 1.575920
C 2.226609 3.795362 2.643079
C -0.143776 4.600124 4.429950
C 2.222881 1.694109 5.348203
C 0.865966 -0.753521 4.392724
C -0.541359 1.183710 6.080324

C -2.511767 -0.708259 3.288338
C -3.927909 1.972986 4.702944
C -5.471532 -0.077896 2.978118
C -3.110564 3.097646 -0.732121
C -5.722897 1.995151 0.099270
C -4.587453 4.406932 1.799279
H -0.297351 1.793247 -1.757568
H 1.710695 1.728353 -0.342066
H -2.096115 -0.037107 -1.441812
H -0.337305 5.108426 1.479568
H -1.654944 3.985775 1.741188
H -0.343484 3.627476 0.570255
H 2.360581 4.829337 2.288909
H 2.818503 3.686440 3.560147
H 2.664009 3.127130 1.885416
H 0.051977 5.658506 4.199357
H 0.389299 4.359433 5.360339
H -1.221293 4.489105 4.623123
H 2.482897 1.136445 6.261625
H 2.097822 2.745629 5.641112
H 3.093654 1.638201 4.678276
H 1.302567 -1.157302 5.322422
H 1.576198 -1.013107 3.594254
H -0.045297 -1.337208 4.222818
H -0.139373 0.698159 6.983621
H -0.704557 2.243436 6.323386
H -1.527925 0.739297 5.885557
H -2.644576 -1.191385 4.268268
H -1.426652 -0.527494 3.245861
H -2.781905 -1.438303 2.514494
H -4.132858 1.409763 5.624341
H -2.990277 2.526768 4.831755
H -4.743144 2.696195 4.556521
H -5.643225 -0.755392 3.828647
H -6.321049 0.619065 2.943732
H -5.490305 -0.677388 2.057861
H -3.612369 3.859881 -1.349978
H -2.103185 3.482532 -0.536492
H -3.018037 2.197166 -1.355534
H -6.196377 2.671891 -0.628823
H -6.469308 1.785286 0.877545
H -5.509983 1.048171 -0.418635
H -5.007127 5.164612 1.122630
H -5.334066 4.183099 2.573671
H -3.696777 4.824139 2.286695
C 0.190902 -0.977078 1.044823
C -0.987556 -0.915335 0.249418
C 1.216356 -0.058889 0.811538
H 0.297351 -1.793247 1.757568
H -1.710695 -1.728353 0.342066
H 2.096115 0.037107 1.441812
U 1.085183 -1.085344 -1.427015
Si -0.409670 -3.415439 -2.957023
Si -0.650942 -0.995450 -4.638419
Si 4.149936 -2.716169 -0.768106
Si 3.852361 -0.814617 -3.163009
N -0.072157 -1.878663 -3.300747
N 3.292475 -1.576657 -1.700009
C 0.567526 -4.036150 -1.575920
C -2.226609 -3.795362 -2.643079
C 0.143776 -4.600124 -4.429950
C -2.222881 -1.694109 -5.348203
C -0.865966 0.753521 -4.392724
C 0.541359 -1.183710 -6.080324
C 2.511767 0.708259 -3.288338
C 3.927909 -1.972986 -4.702944
C 5.471532 0.077896 -2.978118
C 3.110564 -3.097646 0.732121
C 5.722897 -1.995151 -0.099270
C 4.587453 -4.406932 -1.799279
H 0.337305 -5.108426 -1.479568
H 1.654944 -3.985775 -1.741188
H 0.343484 -3.627476 -0.570255
H -2.360581 -4.829337 -2.288909
H -2.818503 -3.686440 -3.560147
H -2.664009 -3.127130 -1.885416
H -0.051977 -5.658506 -4.199357
H -0.389299 -4.359433 -5.360339
H 1.221293 -4.489105 -4.623123
H -2.482897 -1.136445 -6.261625
H -2.097822 -2.745629 -5.641112
H -3.093654 -1.638201 -4.678276
H -1.302567 1.157302 -5.322422
H -1.576198 1.013107 -3.594254
H 0.045297 1.337208 -4.222818
H 0.139373 -0.698159 -6.983621
H 0.704557 -2.243436 -6.323386
H 1.527925 -0.739297 -5.885557
H 2.644576 1.191385 -4.268268
H 1.426652 0.527494 -3.245861
H 2.781905 1.438303 -2.514494
H 4.132858 -1.409763 -5.624341
H 2.990277 -2.526768 -4.831755
H 4.743144 -2.696195 -4.556521
H 5.643225 0.755392 -3.828647
H 6.321049 -0.619065 -2.943732
H 5.490305 0.677388 -2.057861
H 3.612369 -3.859881 1.349978
H 2.103185 -3.482532 0.536492
H 3.018037 -2.197166 1.355534
H 6.196377 -2.671891 0.628823
H 6.469308 -1.785286 -0.877545
H 5.509983 -1.048171 0.418635
H 5.007127 -5.164612 -1.122630
H 5.334066 -4.183099 -2.573671
H 3.696777 -4.824139 -2.286695

References

[1] M. I. Javed, M. Brewer, *Org. Synth.* **2008**, *85*, 189.
[2] L. Merrill, W. A. Bassett, *Rev. Sci. Instrum.* **1974**, *45*, 290.
[3] S. A. Moggach, D. R. Allan, S. Parsons, J. E. Warren, *J. Appl. Crystallogr.* **2008**, *41*, 249.
[4] G. J. Piermarini, S. Block, J. D. Barnett, R. A. Forman, *J. Appl. Phys.* **1975**, *46*, 2774.
[5] S. Parsons, The University of Edinburgh, Edinburgh, UK, **2004**.
[6] a) Bruker-Nonius, Madison, Wisconsin, USA, **2004**, pp. Area; b) A. Dawson, D. R. Allan, S. Parsons, M. Ruf, *J. Appl. Crystallogr.* **2004**, *37*, 410.
[7] Bruker-Nonius, Bruker-AXS, Madison, Wisconsin, USA, **2006**.
[8] G. M. Sheldrick, Bruker-AXS, Madison, WIsconsin, USA, **2004**.
[9] D. J. Watkin, K. Prout, J. R. Carruthers, P. W. Betteridge, R. I. Cooper, Chemical Crystallography Laboratory, University of Oxford, Oxford, UK, **2003**.
[10] M. J. T. Frisch, G. W.; Schlegel, H. B.; Scuseria, G. E.; Robb, M. A.; Cheeseman, J. R.; Scalmani, G.; Barone, V.; Mennucci, B.; Petersson, G. A.; Nakatsuji, H.; Caricato, M.; Li, X.; Hratchian, H. P.; Izmaylov, A. F.; Bloino, J.; Zheng, G.; Sonnenberg, J. L.; Hada, M.; Ehara, M.; Toyota, K.; Fukuda, R.; Hasegawa, J.; Ishida, M.; Nakajima, T.; Honda, Y.; Kitao, O.; Nakai, H.; Vreven, T.; Montgomery, J. A., Jr.; Peralta, J. E.; Ogliaro, F.; Bearpark, M.; Heyd, J. J.; Brothers, E.; Kudin, K. N.; Staroverov, V. N.; Kobayashi, R.; Normand, J.; Raghavachari, K.; Rendell, A.; Burant, J. C.; Iyengar, S. S.; Tomasi, J.; Cossi, M.; Rega, N.; Millam, N. J.; Klene, M.; Knox, J. E.; Cross, J. B.; Bakken, V.; Adamo, C.; Jaramillo, J.; Gomperts, R.; Stratmann, R. E.; Yazyev, O.; Austin, A. J.; Cammi, R.; Pomelli, C.; Ochterski, J. W.; Martin, R. L.; Morokuma, K.; Zakrzewski, V. G.; Voth, G. A.; Salvador, P.; Dannenberg, J. J.; Dapprich, S.; Daniels, A. D.; Farkas, Ö.; Foresman, J. B.; Ortiz, J. V.; Cioslowski, J.; Fox, D. J. , Gaussian 09, Revision C.01, Gaussian, Inc., Wallingford CT, **2009**.
[11] a) X. Y. Cao, M. Dolg, *Journal of Molecular Structure-Theochem* **2004**, *673*, 203; b) X. Cao, M. Dolg, H. Stoll, *J. Chem. Phys.* **2003**, *118*, 487.
[12] a) J. P. Perdew, K. Burke, M. Ernzerhof, *Physical Review Letters* **1997**, *78*, 1396; b) J. P. Perdew, K. Burke, M. Ernzerhof, *Phys. Rev. Lett.* **1996**, *77*, 3865.
[13] T. A. Keith, T. K. Gristmill, **2013**, p. http://aim.tkgristmill.com.
[14] E. D. Glendening, J. K. Badenhoop, A. E. Reed, J. E. Carpenter, J. A. Bohmann, C. M. Morales, C. R. Landis, F. Weinhold, Theoretical Chemistry Institute, University of Wisconsin, Madison WI, **2013**.
